# Scalable Dual-Fluorescence Assay for Functional Interpretation of HNF-4α Missense Variants

**DOI:** 10.3389/fendo.2022.812747

**Published:** 2022-02-14

**Authors:** Yiming Guo, Jing Zhao, Rong Huang, Tao Xu, Kaixin Zhou, Li Zheng

**Affiliations:** ^1^ College of Life Sciences, the University of Chinese Academy of Sciences, Beijing, China; ^2^ Guangzhou Laboratory, Guangzhou, China; ^3^ Key Laboratory of RNA Biology, Center for Big Data Research in Health, Institute of Biophysics, Chinese Academy of Sciences, Beijing, China; ^4^ Shandong First Medical University & Shandong Academy of Medical Sciences, Jinan, China

**Keywords:** HNF-4α, scalable, dual-fluorescence assay, high-throughput, functional interpretation

## Abstract

**Aim:**

The study aimed to develop a scalable dual-fluorescence assay in cells to enable the functional interpretation of HNF-4α missense variants identified in exome sequencing, which can be used to guide clinical diagnosis.

**Methods:**

Using mOrange2 and GFP fluorescence proteins to track the expression of HNF-4α (HNF-4α-mOrange2) and reporter activity under the control of the HNF-1α promoter (pHNF1A-GFP), respectively, we designed a dual-fluorescence assay to evaluate the expression level, cellular localization, and transcriptional function of HNF-4α simultaneously in live cells. To assess the scalable characteristic of the assay, a small library containing five previously reported mutations and wild-type HNF-4α was constructed. Cells infected with this library were sorted into different populations through fluorescence-activated cell sorting (FACS) according to the transcription activity and expression abundance. Cloning and Sanger sequencing were used to detect the mutations of the different groups. High content screening (HCS) assay was used for the validation of individual mutants in the function and expression point of view.

**Results:**

HNF-4α-mOrange2 exhibited nuclear localization and transactivation capability on the HNF-1α promoter as physical HNF-4α does. The expression of HNF-4α-mOrange2 shows a 6-fold induction of GFP expression compared to the control without HNF-4α-mOrange2, which was significantly abolished by the known loss-of-function mutant M373R. The different performances of wild-type and mutant M373R made them distinguishable in the FACS system, empowering the scalable capability of this assay for classifying large numbers of variants combining functional stratification and sequencing. Further application of the assay in the small library showed that three cell populations were seen grouped as Normal (same transactivation as wild type), Reduced^exp_nor^ (reduced transactivation with normal or higher expression), and Reduced^exp_low^ (reduced transactivation with lower expression). Subsequently, Sanger sequencing showed that wild-type HNF-4α was in the Normal group, two mutations (M373R and G79C) were enriched in the Reduced^exp_nor^ group, and three mutations (C115S, L272P, and F83C) belonged to the Reduced^exp_low^ group. These results were validated by further imaging data using HCS assay for individual mutation.

**Conclusions:**

Our study proposes a scalable and informative approach for the characterization of the variants in HNF-4α genes in a quantitative and high-throughput manner.

## Introduction

HNF-4α belongs to the nuclear receptor (NR) superfamily, which comprises the DNA-binding domain (DBD), ligand-binding domain (LBD), activation function domain (AF), and F domain (F) (1). As an important transcription factor, HNF-4α is potentially involved in the regulation of about 40% of transcripts in the liver and controls nearly 11% of genes in pancreatic islets (2–5). Various deleterious mutants in HNF-4α had been shown to cause autosomal dominantly inherited maturity-onset diabetes of the young (MODY1), diazoxide-responsive hyperinsulinemic hypoglycemia (HH), or the development of carcinomas (6–11). More importantly, MODY1 patients are sensitive to treatment with oral sulfonylureas and proper clinical actions could be taken upon the right diagnosis (12, 13). Genetic testing was also highly recommended for HH in the first week of life as HNF-4α mutations are often the underlining cause (10, 13). Therefore, genetic testing of HNF-4α would be valuable for molecular diagnosis, precision treatment, and family genetic counseling.

With the fast-falling of sequencing cost, the number of cataloged genetic variants in public databases such as the Genome Aggregation Database had increased dramatically. The vast majority of missense variants are rare with only 2% of them having a clinical interpretation in ClinVar (14, 15). Within the HNF-4α gene, more than 300 variants have been reported in ClinVar with over 40% of them considered as variants of uncertain clinical significance (VUS). Given that no mutation hotspots were revealed, more VUS are expected to be found and the functional characterization of these VUS would post a significant challenge to the HNF-4α genetic testing and molecular diagnosis.

Although various *in silico* scoring methods such as SIFT and LRT have been developed to assist the functional interpretation of VUS, consensus was hard to achieve between them and they were rarely in agreement with clinical observations (16, 17). Rigorous functional evaluations of VUS *in vitro* are therefore considered a strong line of evidence for molecular diagnosis according to the current ACMG guideline (18).

So far, the functional characterization of HNF-4α variants has mostly utilized mutant plasmid-based assays to evaluate four readouts including transcription activity, protein abundance, cellular localization, and DNA-binding affinity (19). Although such studies were informative to the molecular diagnosis, the assays required to derive them are time consuming and resource intensive, making it difficult to combine different assays or evaluate multiple mutants simultaneously. Given that more VUS of HNF-4α are expected with the easier access of HNF-4α genetic testing, there is an urgent need to develop a scalable assay that could be used to systematically screen all possible HNF-4α mutants in advance.

Here we set out to develop a high-throughput assay for the functional evaluations of HNF-4α variants. Bearing in mind that the transcriptional activity appears to be a superior readout which may be affected by an abnormal expression level, cellular localization, and DNA-binding affinity, we developed a dual-fluorescence reporter assay which could reliably monitor the transactivation activity of an HNF-4α variant together with its expression level and localization in live cells. To fulfill the requirement of scalability, this assay was designed to classify cells, each of which carries a single mutation according to transactivation activity and expression levels using FACS and subsequent sequence using Sanger or next-generation sequencing to distinguish variants with functional change.

## Materials and Equipment

### Materials

The following reagents were purchased from Gibco: Dulbecco’s modified Eagle’s medium (DMEM), fetal bovine serum (FBS), penicillin G sodium and streptomycin, and TrypLE Express. Lipofectamine 3000 Reagent and OMEM were from Life Technologies (Carlsbad, CA, USA). PLP1, PLP2, and PLP-VSVG were kindly gifted from Dr. Ruirui Kong. pcDNA3.1-Flag-HNF-4α (GenBank: NM_000457.6) was purchased from the Public Protein/Plasmid Library (PPL00188-2c), and pRRL.sin-18.ppt.STAT3-GFP.pre (Addgene, #110495) was from Addgene. PQCXIP, VSVG, and Phit were from our lab. Polybrene was from Sigma (St. Louis, MO, USA). The anti-mCherry antibody (26765-1-AP), anti-GFP antibody (50430-2-AP), and anti-GAPDH antibody (60004-1-Ig) were purchased from Proteintech (Wuhan, China). Anti-HNF4A (sc-374229) was from Santa Cruz (Dallas, TX, USA). The PVDF membrane was from Millipore (Bedford, MA, USA). Enhanced chemiluminescence (ECL) was from Promega (Madison, WI, USA).

### Equipment

An influx cell sorter (BD, Biosciences, San Jose, CA, USA) equipped with solid-state lasers for 488- and 561-nm excitation was used. The Opera Phenix High Content Screening (HCS) System is from PerkinElmer, Waltham, MA. FV1200 is from OLYMPUS (Tokyo, Japan).

## Methods

### Cloning and Construction of Plasmids

Wild-type human HNF-4α cDNA was PCR amplified from pcDNA3.1-Flag-HNF-4α, and the HNF-4α2 site mutation variants c.235G>T (p.Gly79Cys), c.248T>G (p.Phe83Cys), c.344G>C (p.Cys115Ser), c.815T>C (p.Leu272Pro), and c.1118T>G (p.Met373Arg) were introduced by site-directed mutagenesis. Expression vectors for human HNF-4α2-WT or mutants were constructed by ligating cDNA fragments directly into the NotI and BamHI sites of PQCXIP-mOrange2. The pTOP-HNF1A-TATA-GFP (pHNF1A-GFP) reporter was constructed by replacing the four repeats of the M67 sequences on pRRL.sin-18.ppt.STAT3-GFP.pre (STAT3-GFP) with the proximal promoter of human hepatocyte nuclear factor-1α (*HNF-1α*) (-105~+30)) with the following sequence: TGTCCCTCTCCGCTGCCATGAGGCCTGCACTTTGCAGGGCTGAAGTCCAAAGTTCAGTCCCTTCGCTAAGCACACGGATAAATATGAACCTTGGAGAATTTCCCCAGCTCCAATGTAAACAGAACAGGCAGGGGC (20).

### Cell Culture and Transfection

HEK 293T cells were cultured in DMEM supplemented with 10% FBS, 100 units/ml penicillin G sodium, and 100 μg/ml streptomycin. During routine passaging, cells were washed with PBS (pH 7.4) and dissociated with TrypLE Express. For transient transfection, cells were plated and were transfected with plasmids as indicated using Lipofectamine 3000 Reagent.

### Viral Preparation and Infection

For virus packaging, the lentivirus system containing transfer plasmid pHNF1AP1-GFP and packing vectors (PLP1, PLP2, PLP-VSVG) or the retrovirus system containing PQCXIP-HNF-4α, VSVG, and Phit were co-transfected into HEK 293T cells with 4×10^6^ cells in a 100-mm dish using Lipofectamine 3000. Media were replaced 24 h later, and viral supernatants were collected at 48 and 72 h, centrifuged at 1,300 rpm for 5 minutes, passed through a 0.45-μm filter (Millipore), and concentrated by lentivirus concentration solution (YEASEN). The virus should be used with optimal virus volumes (MOI of 0.1–0.2) or frozen at -20°C or -80°C for long-term storage. Cells were infected with the virus in the presence of polybrene at a concentration of 1 µg/ml.

### Analytical Flow Cytometry and FACS

Cells were trypsinized and suspended in DMEM for FACS analysis and sorting (BD Influx). Cells were excited with a 488-nm laser (530/40 emission) and a 561-nm laser (610/20 emission). Data analysis was performed using FlowJo_V10 (Tree Star, Ashland) software.

### Live-Cell Imaging and Fluorescent Quantification

Cells were imaged with the Opera Phenix High Content Screening System in confocal mode with the ×20 water NA 1.0 objectives at 37°C and 5% CO_2_ at the indicated time. The fluorophores were detected with the following excitation and emission (Ex/Em) wavelengths: Hoechst 33342 (405/435–480), GFP (488/500–550), and mOrange2 (561/570–630). Quantification analyses were performed using the Harmony software. For high-resolution imaging, cells were grown on small glass coverslips. Forty-eight hours after transfection, images were viewed using the OLYMPUS FV1200 system. All experiments were performed in triplicate.

### Western Blotting

HEK 293T cells were seeded on 96-well plates with different transfection strategies and lysed by the addition of 1× SDS-PAGE loading buffer (reducing). Equal amounts of protein extracts were separated by SDS-PAGE gel and subsequently transferred to a 0.2-μm PVDF membrane for 1 h at 400 mA and blocked at room temperature for 1 h in 1× TBST (20 Mm Tris base, 0.15 M NaCl, 0.1% Tween-20, 5% milk). Proteins were analyzed using the anti-mCherry antibody, anti-HNF-4α antibody, anti-GFP antibody, and anti-GAPDH antibody. Bands were developed using ECL substrates, and band intensities were quantified by ImageJ software.

### DNA Extraction and Cloning for Sanger Sequencing

Total genome DNA was extracted using phenol-chloroform DNA extraction protocol. The fully integrated HNF-4α sequences were amplified using primers (HNF-4α2-F: 5′-CCGCGGCCGCACCATGCGACTCTCCAAAAC-3′; HNF-4α2-R: 5′-TGGGATCCGGGATAACTTCCTGCTTG-3′) and cloned in the PQCXIP vector for Sanger sequencing.

## Results

### Construction of a Dual-Fluorescence Assay Simultaneously Monitoring the Transcription Activity, Expression, and Localization of HNF-4α in Live Cell

**Figure 1:**
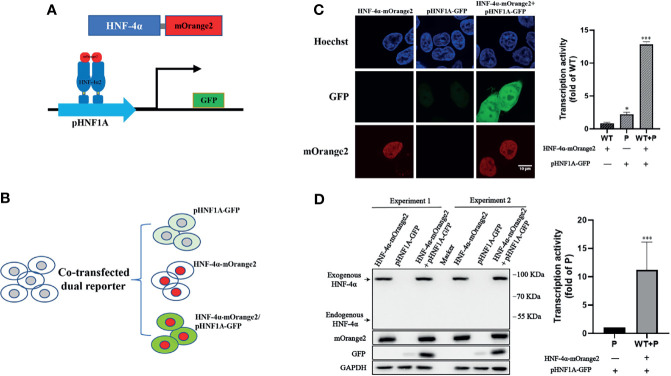
Establishment of the dual-fluorescence assay for functional evaluation on the HNF-4α. **(A)** A schematic diagram of the dual-fluorescence reporter system. The C-terminal of HNF-4α was fused with a red fluorescent protein mOrange2 and was used to track the HNF-4α cell location and expression; pHNF1A was the GFP reporter under control of the HNF-1α promoter which was used to detect the transcription activity of the HNF-4α dimer. **(B)** Overview of the assay working in cell lines. **(C)** Representative confocal microscopy imaging of dual-fluorescence assay and quantification of the transcription activity. Nuclear staining (blue); 488-nm channel for pHNF1A-GFP (green); 561-nm channel for HNF-4α-mOrange2 (red). The GFP intensity of the three groups has also been quantified with three fields in one experiment; the data were analyzed using a Student t-test (*p < 0.05, ***p < 0.001). WT, wild-type HNF-4α-mOrange2, P, pHNF1A-GFP. **(D)** Representative Western blots and quantification of GFP intensity of cells transfected with plasmids as indicated for 48 h. The anti-mCherry antibody and anti-HNF-4α antibody were used for detection of mOrange2-conjugated HNF-4α proteins and HNF-4α proteins, respectively. The anti-GAPDH antibody was the internal reference in this experiment. The anti-GFP antibody showed the transcription activity. Data represented as mean± SD from six independent experiments.

To design a dual-fluorescence reporting system monitoring the expression level and transactivation activity of HNF-4α in live cells, we constructed a GFP reporter under the control of the HNF1A promoter (pHNF1A-GFP) and introduced the mOrange2 fluorescence protein to the C terminal of HNF-4α (HNF-4α-mOrange2) (**Figure 1A**). As HEK 293T cells have an undetectable endogenous HNF-4α expression (**Figure 1D**), the GFP reporter was expected to express only in the presence of transcript factor HNF-4α-mOrange2 (**Figure 1B**). As expected, co-transfection of the GFP reporter and wild-type HNF-4α-mOrange2 led to about 6-fold higher GFP expression compared to the cells transfected with the GFP reporter alone. In addition, confocal imaging showed the nuclear localization of HNF-4α-mOrange2 (**Figure 1C**). The correct expression was also evidenced by Western blot using a specific antibody (**Figure 1D**). This result demonstrates that exogenous HNF-4α-mOrange2 could work as that of endogenous HNF-4α does with proper expression, cellular distribution, and transactivation on the HNF-1α promoter in the HEK 293T cell line.

**Figure 2:**
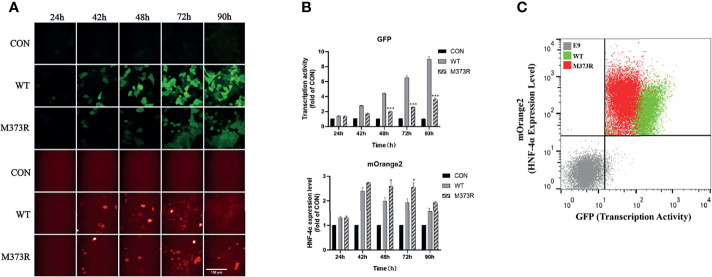
Optimization of the assay in the single-cell clone HEK293T_pHNF1AP_E9 stably expressing the pHNF1A-GFP reporter. **(A, B)** Kinetic performance of this assay in HEK293T_pHNF1AP_E9 cells transiently transfected with HNF-4α or mutant M373R. Data were the means of triplicate fields from one experiment and were conducted twice independently; error bars show s.d. *p < 0.05, ***p < 0.001. Scale bar: 50 μm. **(C)** Sorting of HEK293T_pHNF1AP_E9 (E9) cells expressing wide-type HNF-4α (WT, green color) or M373R (M373R, red color) populations, negative control: HEK293T_pHNF1AP_E9 (gray color). The transcription activity of mutant M373R was significantly lower than that of wild type, p < 0.0001.

In order to improve the performance of the dual-fluorescence reporter, we further established cell lines stably expressing the pHNF1A-GFP reporter with the lentivirus system in the HEK 293T cell line, upon which the E9 clone was selected for the highest specificity for exogenous wild-type HNF4A transactivation. Compared to the HEK293T_pHNF1AP_E9 cells without HNF-4α-mOrange2 expression, cells transiently transfected with wild-type HNF-4α-mOrange2 exhibit a significantly increased GFP expression, which was initiated from 24 h after transient transfection and reached the highest about 90 h later. The system was validated with a loss-of-function mutant identified in HH and MODY1 (11, 19), M373R, which showed the same expression pattern with reduced transactivation (**Figures 2A, B**). The transactivation activity difference between wild-type and M373R mutant was significant and distinguishable with FACS (p < 0.0001; **Figure 2C**). These results demonstrate that we had established a dual-fluorescence reporting system in the HEK 293T cell line for further distinguishing various unknown mutants according to the effects on the transactivate ability and expression level.

### Validation of the Assay With a Library Containing Wild-Type HNF-4α and Five Previously Reported Loss-of-Function Mutations

**Figure 3:**
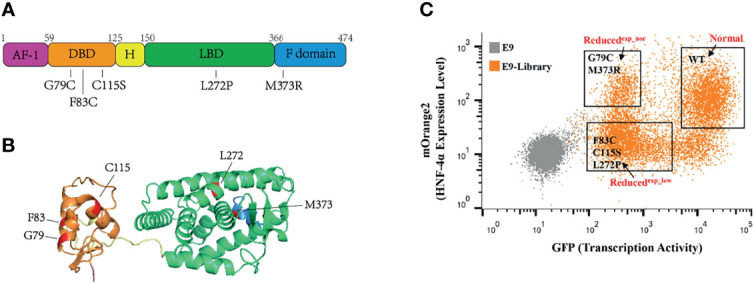
Application of this assay in a small library containing wild-type HNF-4α and five mutants. **(A)** Schematic representation of the HNF-4α (NM_000457.6) structure, and distribution of single amino acid mutations used in this study. Five main functional domains: AF-1, activation function 1 (amino acids 1–59), purple color; DBD, DNA-binding domain (amino acids 60–125), orange color; H, hinge domain (amino acids 126–150), yellow color; LBD, ligand-binding domain (amino acids 151–366), green color; F domain (amino acids 367–474), blue color. **(B)** Three-dimensional model of human HNF-4α (4iqr) with schematic function domains of HNF-4α and distribution of five deleterious mutations used in this study. The mutations were highlighted in red. Four functional domains: the DBD, orange color; H domain, yellow color; LBD, green color and F domain, blue color. **(C)** Gating of the HEK293T_pHNF1AP_E9(E9) cells infected with library into three distinct populations based on the GFP and mOrange2 level. The mutants labeled in the gates were identified through cloning and Sanger sequence.

To validate this assay, we construct a small library by equally mixing wild-type HNF-4α with five previously reported loss-of-function mutations, including three mutations (G79C, F83C, C115S) in the DBD, one mutation (L272P) in the LBD, and one mutation (M373R) in the F domain (**Figures 3A, B**). HEK293T_pHNF1AP_E9 cells (4 × 10^6^) in a 10-cm dish were incubated with retrovirus packaged with the library at an MOI of 0.1 to ensure each cell was infected with only one virus. Selected with puromycin for 2 doubling times, cells were subsequently sorted according to the transcription activity and expression level by FACS. Three cell populations were collected which were named as Normal (same transactivation as wild type), Reduced^exp_nor^ (reduced transactivation with normal or higher expression), and Reduced^exp_low^ (reduced transactivation with lower expression) (**Figure 3C**). Integrated cDNAs were cloned into pQCXIP-mOrange2. We randomly selected five clones each group for Sanger sequencing. The results showed that two mutations (M373R and G79C) are enriched in the Reduced^exp_nor^ group, three mutations (C115S, L272P, and F83C) belong to the Reduced^exp_low^ group, while the wild type belong to the Normal group.

**Figure 4:**
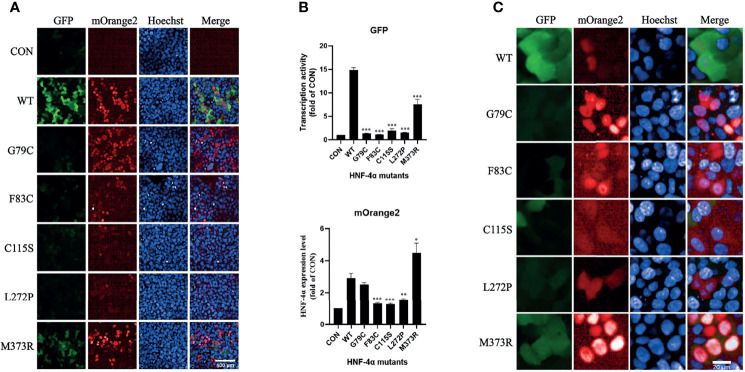
Validation of the library mutants individually in HCS with confocal mode. **(A)** Evaluation of the transcription activity, expression level, and localization of HNF-4α wild type or mutants using the HCS image. HEK293T_pHNF1AP_E9 cells transfected with wild-type HNF-4α or different mutants as indicated for 72 h. GFP corresponds to the transactivists’ ability, and mOrange2 represents the expression of HNF-4α or mutants. Scale bar: 100 μm. **(B)** Quantitative of the transcription activity and expression level of all the plasmids in the library we used. Shown is the fold change of GFP and HNF-4α-mOrange2 intensity. All data were presented as mean ± SD (n = 3 fields, and experiments were conducted twice independently. Statistical significance between WT and mutants (G79C, F83C, C115C, L272P, M373R) is indicated as asterisks: *p < 0.05, **p < 0.01, ***p < 0.001 (Student’s t-test). **(C)** Cellular localization of the wild type or mutants of HNF-4α. Representative cells were shown except for three mutants (F83C, C115C, and L272P) which cells with higher mOrange2 intensity were selected for more details. Scale bar: 20 μm.

To verify the result, we tested the mutants individually using this dual-fluorescence assay in high content screening assay (HCS). Three mutants (F83C, C115S, and L272P) classified as Reduced^exp_low^ group with high confidence were found significantly repressing the transcription activity with lower expression, and two mutants (G79C and M373R) classified as Reduced^exp_nor^ group were found significantly disrupting the transcription activity with normal or higher expression (**Figures 4A, B**). The effects of all the missense variants on the transactivation activity and expression level were consistent with the results from FACS. In addition, we found that the localization of two mutants (G79C and M373R) was predominantly in the nucleus. By contrast, the localization of the other three mutants (F83C, C115S, and L272P) exhibited more diffusion in the cytoplasm compared to the wild-type HNF-4α (**Figure 4C**), which may partly contribute to the loss of function and lower abundance. In conclusion, our results clearly showed that this dual-fluorescence assay could be used as a simple, precise, and informative assay to evaluate the effect of mutations of HNF-4α.

## Discussion

In this study, we firstly developed a dual-fluorescence reporter assay that could simultaneously examine HNF-4α variants on three mechanistic aspects including the transcriptional activity, protein abundance, and cellular localization. Then the assay was validated with a small library of five established loss-of-function variants with wild-type HNF-4α to demonstrate its reliability and the potential for high-throughput functional screening of VUS in HNF-4α.

The five mutants used to validate our assay were selected to represent the wide spectrum of potential functional defects that could originate from many possible HNF-4α variants. G79C and F83C in the DNA-binding domain had been reported with reduced DNA-binding affinity (6). M373R in the F domain was reported to impair the HNF-4α function by interrupting its interactions with coactivators without affecting DNA-binding affinity (19). C115S in the DBD and L272P in the LBD were identified in HH patients with severe phenotypes but poorly characterized function (10). In addition, the other three variants were also reported with a direct role in the pathogenesis of disease outcomes (6, 19). Therefore, these five mutants from different functional domains of HNF-4α, with various functional defects and all resulting in clinical consequences, could solidly anchor the assay for the examination of other VUS within this gene.

The exogenous wild-type HNF-4α-mOrange2 showed efficient regulation on the HNF-1α promoter, and our assay found that all the five established mutants had consistent and significant loss of transcriptional activity. This indicated that our *in vitro* system could mimic the action of physiological HNF-4α and provide reliable evidence of transcriptional activity on VUS.

While examining the HNF-4α protein abundance, our assay demonstrated the ability to distinguish variants with varying degrees of expression levels. Of the five loss-of-function variants, G79C and M373R were normally expressed while F83C, C115S, and L272P showed a lower expression. In regard to the cellular localization, we showed that three mutants with a lower expression exhibited more diffusion in the whole cells compared to the wild type and other two mutants. It was reported that the phosphorylation on the S87 in the DBD domain and Y286/Y288 in LBD could enhance nuclear export and cytoplasmic aggregation which induced proteasomal degradation (21, 22). Therefore, we propose that the reasons for those variants to exhibit low protein abundance in our assay might be due to enhanced degradation through posttranslational modifications such as phosphorylation (22–25). In addition, it is worth noting that the expression level of M373R was higher in our assay but was previously reported with reduced HNF-4α protein stability and in turn low abundance (19). One possible explanation for the discrepancy was that our assay was relatively simple as compared to the previous study which was complicated and might have a larger chance to generate more artificial results. Such a possibility was further strengthened as M373R showed a higher expression when tested individually (**Supplementary Figure 1**). Taken together, these results demonstrated that our assay was capable of monitoring the protein abundance and cellular localization of HNF-4α VUS.

One important feature of our assay was that it utilized FACS to examine multiple functions of HNF-4α variants simultaneously in live cells. The assay would be able to differentiate a spectrum of variants with varying levels of functional loss in either transcription activity or protein abundance. The small validation library of five variants demonstrated that after sorting the cells, sequencing could be used to classify or quantify the functional loss of each variant from a mixed pool of cells carrying different mutants. Therefore, our assay paves the way to conduct high-throughput functional screening of a saturated library of HNF-4α mutants and provide a “lookup table” for functional annotation of VUS.

One limitation of our assay was that the DNA-binding affinity was not measured directly. However, the transcription activity level test in the assay could partially capture such defect. On the other hand, our assay had a clear intention to evaluate the functional impact of HNF-4α mutants relevant to MODY1. That is why we opted to choose HNF1A expression as the reporter of HNF-4α transcriptional activity instead of other targets. Furthermore, we selected the 293T cell line to evaluate HNF-4α function because of the high expression efficiency of exogenous plasmids and undetectable endogenous HNF-4α as compared to INS-1 cells. This assay could be further improved by replacing the reporter or cell line.

In conclusion, our dual-fluorescence assay could reliably evaluate multiple functional impacts of HNF-4α variants related to MODY1. The successful validation with a small library of five loss-of-function variants demonstrated the potential of this assay to be applied in high-throughput screening of HNF-4α variants. Systematically characterizing the functional impacts of all HNF-4α variants would arm the genetic consultants and clinicians with valuable prior knowledge when facing carriers of HNF-4α VUS.

## Data Availability Statement

The original contributions presented in the study are included in the article/**Supplementary Material**, further inquiries can be directed to the corresponding authors.

## Author Contributions

YG and LZ participated in the design and analysis and wrote the manuscript. JZ and RH contributed to the data measurement and analysis. TX and KZ participated in the design and revision. LZ and KZ wrote the manuscript with help from all authors. All authors read the manuscript and discussed the interpretation of results. All authors contributed to the article and approved the submitted version.

## Funding

This work was supported by grants from the National Natural Science Foundation of China (31570765), the National Key R&D Program of China (2018YFC2001003), and the Strategic Priority Research Program of the Chinese Academy of Sciences (XDB38020100). The funding agency had no role in the design of the study; in the collection, analyses, or interpretation of data; in the writing of the manuscript; or in the decision to publish the results.

## Conflict of Interest

The authors declare that the research was conducted in the absence of any commercial or financial relationships that could be construed as a potential conflict of interest.

## Publisher’s Note

All claims expressed in this article are solely those of the authors and do not necessarily represent those of their affiliated organizations, or those of the publisher, the editors and the reviewers. Any product that may be evaluated in this article, or claim that may be made by its manufacturer, is not guaranteed or endorsed by the publisher.
