# Supplementary material for: Scalable Dual-Fluorescence Assay for Functional Interpretation of HNF-4α Missense Variants
Source: Front Endocrinol (Lausanne). 2022 Feb 14;13:812747. doi: 10.3389/fendo.2022.812747 (PMC8883583; doi:10.3389/fendo.2022.812747)
Supplement: Supplementary file 1 [file DataSheet_1.docx]

Supplementary Material

**Supplementary Material**

D- luciferin(L9504) was purchased from Sigma. PGL3-Basic and pEGFP-N1were from our lab.

**Luciferase reporter assay.**

The PGL3-pHNF1A reporter was constructed by ligating the Proximal promoter of HNF-1α（-105 ~+30）directly into the XhoⅠand Hind Ⅲ sites of PGL3-Basic plasmid. To analyze HNF-1α promoter activity, 0.1 ug of PQCXIP- HNF-4α, 0.1 ug PGL3-pHNF1A, and 0.02 ug pEGFP-N1 plasmid were co-transfected into 96-well HEK293T cells. Luminescence was measured 48h post-transfection using a Luciferase assay system. Briefly, cells were washed in PBS and were added 100 ul of PBS containing 5ug/ml luciferin, and luciferase activity was measured immediately on a plate reader. The EGFP-N1 plasmid was used to determine the transfection efficiency. Each HNF-4α mutant was tested in three independent transfection experiments.

**Results**

Mutation M373R showed a higher expression level while reduced transactivation when tested individually in HEK293T cells using luciferase reporter assay, with pEGFP-N1 co-transfected as an indicator of transcription efficiency.

**Supplementary Figures**


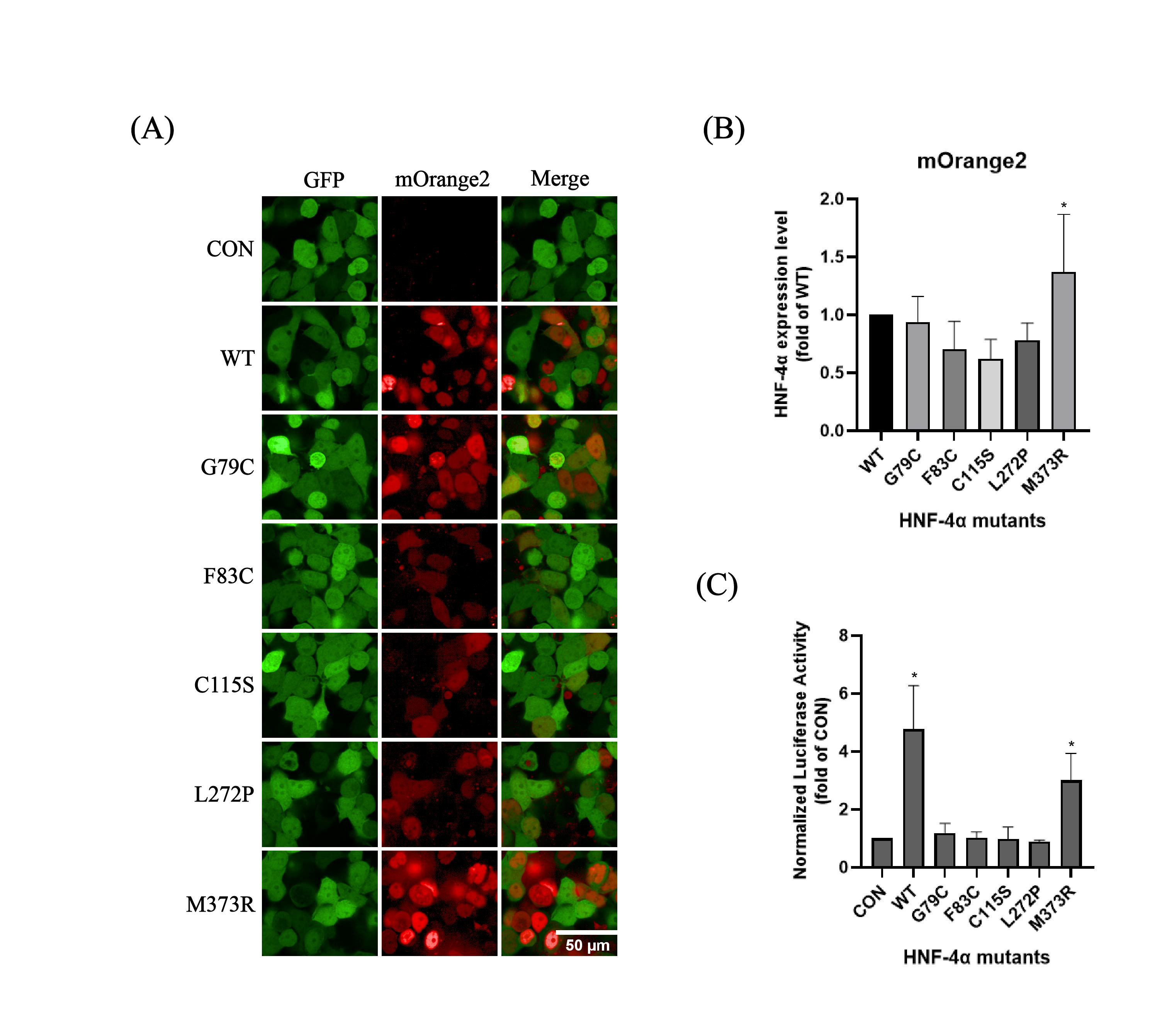


**Supplementary Figure 1.** Characteristic of mutants using HCS and luciferase assay. **(A)** HEK293T cells were transiently transfected with PGL3-pHNF1A (-105 ~+30) and different plasmids as indicated for 48 hours. pEGFP-N1 was co-transfected as an indicator of transcription efficiency. Images were obtained with HCS in a confocal mode. Scale bar: 50 μm. **(B and C)** Quantitative of the expression level(**B**) and transcription activity(**C**) of the mutants as indicated. All data were presented as mean ± SD. N=3, *p<0.05 vs WT group**(B)** and *p<0.05 vs CON group**(C)**.
